# N-Ethylmaleimide Sensitive Factor (NSF) Inhibition Prevents Vascular Instability following Gram-Positive Pulmonary Challenge

**DOI:** 10.1371/journal.pone.0157837

**Published:** 2016-06-29

**Authors:** Ji Young Lee, Helena M. Linge, Kanta Ochani, Ke Lin, Edmund J. Miller

**Affiliations:** 1 The Elmezzi Graduate School of Molecular Medicine, Manhasset, New York, United States of America; 2 The Center for Heart and Lung Research, The Feinstein Institute for Medical Research, Manhasset, New York, United States of America; 3 Hofstra North Shore-LIJ Medical School, Hempstead, New York, United States of America; University of Alabama at Birmingham, UNITED STATES

## Abstract

**Background:**

The Acute Respiratory Distress Syndrome (ARDS), remains a significant source of morbidity and mortality in critically ill patients. Pneumonia and sepsis are leading causes of ARDS, the pathophysiology of which includes increased pulmonary microvascular permeability and hemodynamic instability resulting in organ dysfunction. We hypothesized that N-ethylmaleimide sensitive factor (NSF) regulates exocytosis of inflammatory mediators, such as Angiopoietin-2 (Ang-2), and cytoskeletal stability by modulating myosin light chain (MLC) phosphorylation. Therefore, we challenged pulmonary cells, *in vivo* and *in vitro*, with Gram Positive bacterial cell wall components, lipoteichoic acid (LTA), and peptidoglycan (PGN) and examined the effects of NSF inhibition.

**Methods:**

Mice were pre-treated with an inhibitor of NSF, TAT-NSF700 (to prevent Ang-2 release). After 30min, LTA and PGN (or saline alone) were instilled intratracheally. Pulse oximetry was assessed in awake mice prior to, and 6 hour post instillation. *Post mortem*, tissues were collected for studies of inflammation and Ang-2. *In vitro*, pulmonary endothelial cells were assessed for their responses to LTA and PGN.

**Results:**

Pulmonary challenge induced signs of airspace and systemic inflammation such as changes in neutrophil counts and protein concentration in bronchoalveolar lavage fluid and tissue Ang-2 concentration, and decreased physiological parameters including oxygen saturation and pulse distention. TAT-NSF700 pre-treatment reduced LTA-PGN induced changes in lung tissue Ang-2, oxygen saturation and pulse distention. *In vitro*, LTA-PGN induced a rapid (<2 min) release of Ang-2, which was significantly attenuated by TAT-NSF700 or anti TLR2 antibody. Furthermore, TAT-NSF700 reduced LTA-PGN-induced MLC phosphorylation at low concentrations of 1–10 nM.

**Conclusions:**

TAT-NSF700 decreased Ang-2 release, improved oxygen saturation and pulse distention following pulmonary challenge by inhibiting MLC phosphorylation, an important component of endothelial cell retraction. The data suggest that inhibition of NSF in pneumonia and sepsis may be beneficial to prevent the pulmonary microvascular and hemodynamic instability associated with ARDS.

## Introduction

Acute Respiratory Distress Syndrome (ARDS) occurs annually in around 190,000 individuals in the U.S. and remains a significant source of morbidity and mortality in critically ill patients [1]. The respiratory failure tends to be severe enough to require ventilatory support in approximately 80% of cases [2]. Pneumonia and sepsis are leading causes of ARDS, the pathophysiology of which includes increased pulmonary microvascular permeability and hemodynamic instability resulting in organ dysfunction.

A common feature of both severe sepsis and ARDS is an excessive uncontrolled systemic inflammation and endothelial dysfunction presented as a ‘cytokine storm’ [3]. The outcome of this ebullient response is the development of increasing vascular leakage leading to extravascular fluid accumulation, intravascular volume depletion, circulatory and respiratory failure. These changes lead to an imbalance between an increased oxygen demand as a result of increased cellular metabolism, and decreased oxygen transport. Decreased oxygen transport is in part due to a combination of myocardial depression and inefficient oxygen extraction related to changes in the peripheral microvasculature [4, 5]. Involved organs such as lungs undergo diffuse inflammation that results in loss of endothelial barrier integrity.

Endothelial cells play an important role in vascular stability and homeostasis. In sepsis, endothelial cells are ‘activated’ and undergo a series of functional and structural changes including secretion of various inflammatory mediators and changes in endothelial barrier permeability [6]. Endothelial cells have specific vesicular structures such as Weibel-Palade bodies (WPBs) which can store pre-synthesized molecules and secrete them when endothelial cells are activated. Compartmentalization and selective secretion of different molecules by WPBs have been described [7].

So far, therapeutic strategies targeting molecular pathways have been either too broad (general anti-inflammatory) or too narrow (anti-single cytokine therapy), and their results have been disappointing [8]. Considering inflammation as a necessary process in clearing invading organisms, completely blocking it may be equally detrimental. Thus, it may be ideal if the therapy can be tailored to support antimicrobial effects while targeting a group of molecules that are involved in the most critical and characteristic pathologic process occurring in sepsis: vascular dysfunction.

Our study focused on the role of Angiopoietin-2 (Ang-2), a member of the angiopoietin/tyrosine kinase family with immunoglobulin-like and EGF-like domains (Tie) system. Ang-2 modulates vascular permeability, particularly in inflammatory lung disease, sepsis and ARDS. Ang-2 is constitutively synthesized and stored in the vesicles in endothelial cells and released rapidly by exocytosis upon inflammatory stimuli [9]. Its role in endothelial cell barrier disruption in sepsis has been well described [10–12], and is mediated by increased myosin light chain phosphorylation (MLC-p) [9], and there is a correlation between Ang-2 and severity and mortality [13, 14].

Ang-2 is stored in intracellular vesicles and its release, from endothelial cells, to the extracellular compartment is mediated by *N-*ethylmaleimide-sensitive factor (NSF) [15]. We hypothesized that uncontrolled excessive secretion of Ang-2 and MLC phosphorylation are mediated by NSF which is responsible for vascular instability in sepsis/ARDS. Here we show that modifying Ang-2 secretion and MLC phosphorylation, by NSF inhibition, significantly improves pulmonary and cardiovascular outcomes following pulmonary challenge, and explore the mechanism of action *in vitro*.

## Materials and Methods

### Animal model

All animal experiments received prior approval by the Institutional Animal Care and Use Committee of The Feinstein Institute. We used a mouse model of Gram positive non-progressive ALI, as we have described previously [16]. Male Balb/c mice, 8–10 weeks old (n = 8/group) were pre-treated *i*.*p*. with the NSF inhibitor, TAT-NSF700 (0.5mg/kg in 125μl, (AnaSpec, Fremont, CA)) or with an equal volume of the vehicle (saline). After 30min, the mice were anesthetized (2% isoflurane with 98% oxygen) the trachea was surgically exposed, and cell wall components from gram positive bacteria lipoteichoic acid (LTA; 150μg) and peptidoglycan (PGN; 500μg) (Sigma, St. Louis, MO) in 50μl sterile saline, or an equal volume of saline alone, were instilled intra-tracheally using a 29 gauge needle. The incision was sutured and the animals were allowed to recover on a heated blanket for 20 min. The mice were then maintained at ambient temperature with food and water *ad libitum*. Neck collar clip pulse oximetry (Starr Life Sciences Corp., Allison Park, PA) was used to assess oxygen saturation, respiratory rate, pulse distention and heart rate in awake mice prior to, and 6 hour post instillation. Mice were euthanized by exsanguination, via cardiac puncture, under a surgical plane of anesthesia. Post mortem, bronchoalveolar lavage of the lungs was performed using 3 infusions of 0.6 ml saline each. The bronchoalveolar lavage fluid (BALF). BALF and plasma was stored at -80°C until analysis. The lungs were then excised and stored at -80°C, for Ang-2 analysis.

### Plasma, tissue homogenates and BAL fluid analysis

Immediately after collection of BAL fluids, erythrocytes were lysed using 0.2% saline and the remaining cells were resuspended in Hanks' Balanced Salt Solution (Invitrogen, Grand Island, NY). Total cell count of each BAL sample was determined using a Neubauer hemocytometer (Hausser Scientific, Horsham, PA). Differential cell counts were performed (200 cells for each experimental condition) on cytospin slides stained with Protocol HEMA3 solution (Fisher scientific, Fair Lawn, NJ). MPO levels were determined using Suzuki’s protocol [17]. Total protein concentration in BAL fluid was measured using Coomassie protein assay kit (Thermo Scientific, Rockford, IL). Ang-2, KC and MIP-2 concentration in plasma, tissue homogenates and BAL fluid was measured using specific ELISAs (Ang-2, R&D Systems, Minneapolis, MN; KC and MIP-2, Millipore Inc., Billerica, MA) according to the manufacturer’s instructions.

### Cell culture

The human pulmonary microvascular endothelial cell line, HPMEC-ST1.6R (kindly provided by C. James Kirkpatrick, Institute of Pathology, Johannes-Gutenberg University, Germany), was cultured in Endothelial Cell Growth Medium MV2 (Promocell, Germany) to confluence. The cells were then incubated overnight in serum free medium, Medium 199 with Earle's BSS (Lonza Biowhittaker, Walkersville, MD). The following day, cells were pre-treated with specific pathway inhibitors, TAT-NSF700 or anti TLR2 inhibitor (Biolegend, Sandiego, CA) for 1h before being incubated with cell wall components of Gram-positive bacteria LTA and PGN (Sigma-Aldrich, St. Louis, MO) for 2min-24h. For constitutive secretion assessment, cells were incubated with inhibitors of pathway components for 24h. Culture medium was collected and Ang-2 levels were determined by using above mentioned specific ELISA.

### Western blot

Cells were washed twice with cold (4°C) PBS buffer, cells were lysed in lysis buffer containing 150 mM NaCl, 50 mM Tris-HCl (pH 7.4), 2 mM EDTA (pH 8.0), 1% octylphenol polyethyleneglycol (NP-40), 0.5% deoxycholic acid, 0.1% SDS, and 1 mM PMSF. Lysates were separated from debris by centrifugation (7800 × g) for 15 min, and lysates were boiled for 5 min in Laemmli sample buffer (Bio-Rad Laboratories, Hercules, CA). Proteins (total protein content was 15 μg/lane) were separated by sodium dodecyl sulfate polyacrylamide gel electrophoresis (SDS-PAGE), and transferred to a polyvinylidine difluoride membrane (Millipore, Billerica, MA). Prestained molecular weight standards (Crystalgen Inc., Plainview, NY) were run with each gel to determine the approximate molecular weight of detected bands. Then membranes were incubated with the specific primary antibodies (phospho-MLC, 1:1000 and phospho-ERK, 1:2000, Cell Signaling, Danvers, MA; total ERK (ERK1/2), 1:1000, Santa Cruz Biotechnology, Dallas, TX) and fluorescently-labeled secondary antibodies (1:10,000 dilution, LI-COR, Lincoln, NE). After washing with tris-buffered saline contained 0.1% tween-20 (TBS-T) protein bands were quantified by LI-COR Odyssey scanner.

### Statistics

All values were expressed as mean ± standard deviation (SD). Multiple groups were compared using analysis of variance (ANOVA) using the Dunnett's post hoc test. Results were considered statistically significant at p < 0.05.

## Results

### *In Vivo* Studies

#### TAT-NSF700 attenuated LTA-PGN induced Ang-2 secretion and oxygen desaturation following pulmonary challenge

Although ARDS has many etiologies (including trauma, aspiration, pancreatitis, and transfusion), pneumonia and sepsis remain the leading causes. The clinical manifestations of ARDS include severe hypoxemia with acute onset and bilateral infiltrates. We have developed an animal model in which components of the Gram positive bacterial cell wall LTA-PGN are instilled intra-tracheally inducing local lung inflammation including histologic changes, increased neutrophil recruitment, MPO activity and protein levels in BALF in a dose dependent manner [16]. The model also demonstrates increased lung edema shown by increased lung wet-to-dry ratio and signs of systemic inflammation including increased plasma cytokine concentration and changed heart rate, respiratory rate, pulse distention and oxygen saturation [18]. We used this model to assess the effects of TAT-NSF700. Three groups were used: saline, LTA-PGN, and TAT-NSF700 (n = 8/group). Baseline hemodynamic parameters were measured in all three groups and there were no significant differences among groups. The TAT-NSF700 group were then treated i.p. with the NSF inhibitor, whilst the other two groups received an equal volume of saline. After 30 minutes LTA-PGN was intratracheally instilled for LTA-PGN and TAT-NSF700 groups and saline was instilled for control group.

We hypothesized that sepsis related systemic inflammation and hemodynamic instability are consequences of excessive secretion of various inflammatory mediators and cytoskeletal destabilization. Thus, by blocking a transporter involved in the secretory process we expected to see decreased disease severity. Studies have shown that NSF is an important mediator of the trafficking involved in exocytosis of vesicles containing inflammatory mediators [19, 20], and it can be inhibited by TAT-NSF700, a synthetic peptide inhibitor of NSF [21].

**Fig 1:**
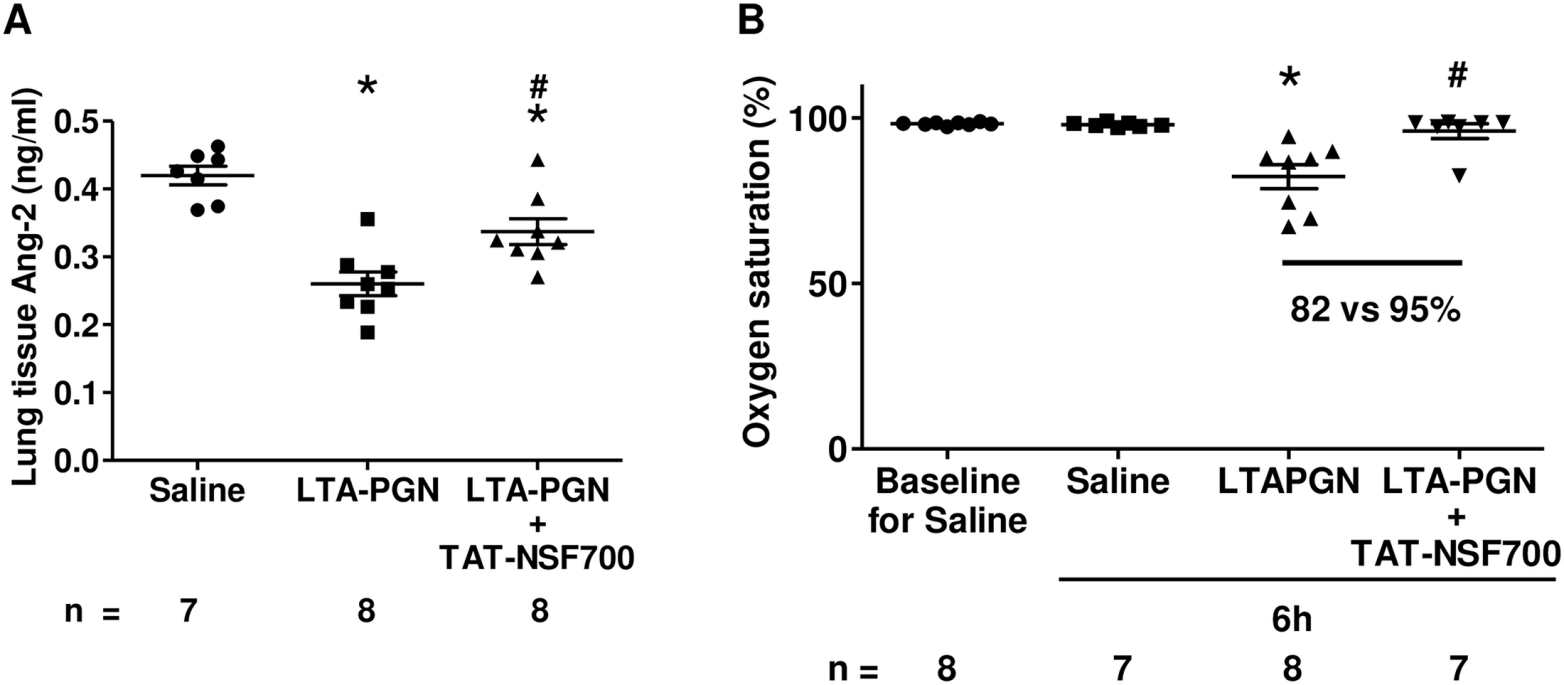
TAT-NSF700 effects on LTA-PGN induced lung Ang-2 level change and oxygen desaturation. Male Balb/c (n = 8/group) were pre-treated with an NSF inhibitor TAT-NSF700 or saline *i*.*p*. After 30min, mice were anesthetized and LTA-PGN or saline alone were instilled intra-tracheally. Pulse oximetry and other parameters (respiratory rate, pulse distention and heart rate, the results will be demonstrated in Fig 2) were assessed in awake mice prior to, and 6 hour post instillation. Then, mice were anesthetized and, euthanized by exsanguination via cardiac puncture. BAL was performed and the lungs were excised and stored at -80°C. During the experiment, one control group mouse died immediately after initial tracheal instillation and one TAT-NSF700 group mouse died around 6 hours after the surgery. In both cases, the death is thought to be from surgical complications. **[A]** Ang-2 levels were measured in 6h post tracheal instillation lung tissue homogenates by ELISA. **[B]** Oxygen saturation at baseline and 6 hours post LTA-PGN instillation is demonstrated (each column shows mean ± standard error; n = total number of wells; * *p* <0.05 *versus* Saline group; # *p* <0.05 *versus* LTA-PGN group).

To assess the effect of TAT-NSF700 on inhibiting secretion of Ang-2, we measured lung tissue Ang-2 levels 6 hours post-LTA-PGN tracheal instillation. Compared to a saline group, lung tissue homogenate Ang-2 was decreased in LTA-PGN group (Fig 1A). TAT-NSF700 pre-treatment significantly attenuated LTA-PGN induced Ang-2 level change, suggesting that TAT-NSF700 has an inhibitory effect on LTA-PGN induced Ang-2 secretion from the lung.

On neck collar clip pulse oximetry measurement, there was a reduction in oxygen saturation in the LTA-PGN treated group (Fig 1B). However, there was no statistically significant decrease in oxygen saturation in those animals that received the TAT-NSF700 (82 ± 10 vs 95 ± 5%).

#### TAT-NSF700 prevented LTA-PGN induced pulse distention change; a potential mechanism of improvement in oxygen saturation

To explain TAT-NSF700 effect on oxygen saturation in physiologic standpoint, we considered three major etiologies of hypoxemia that are applicable in our experimental setting; shunt, hypoventilation and ventilation-perfusion mismatch.

**Fig 2:**
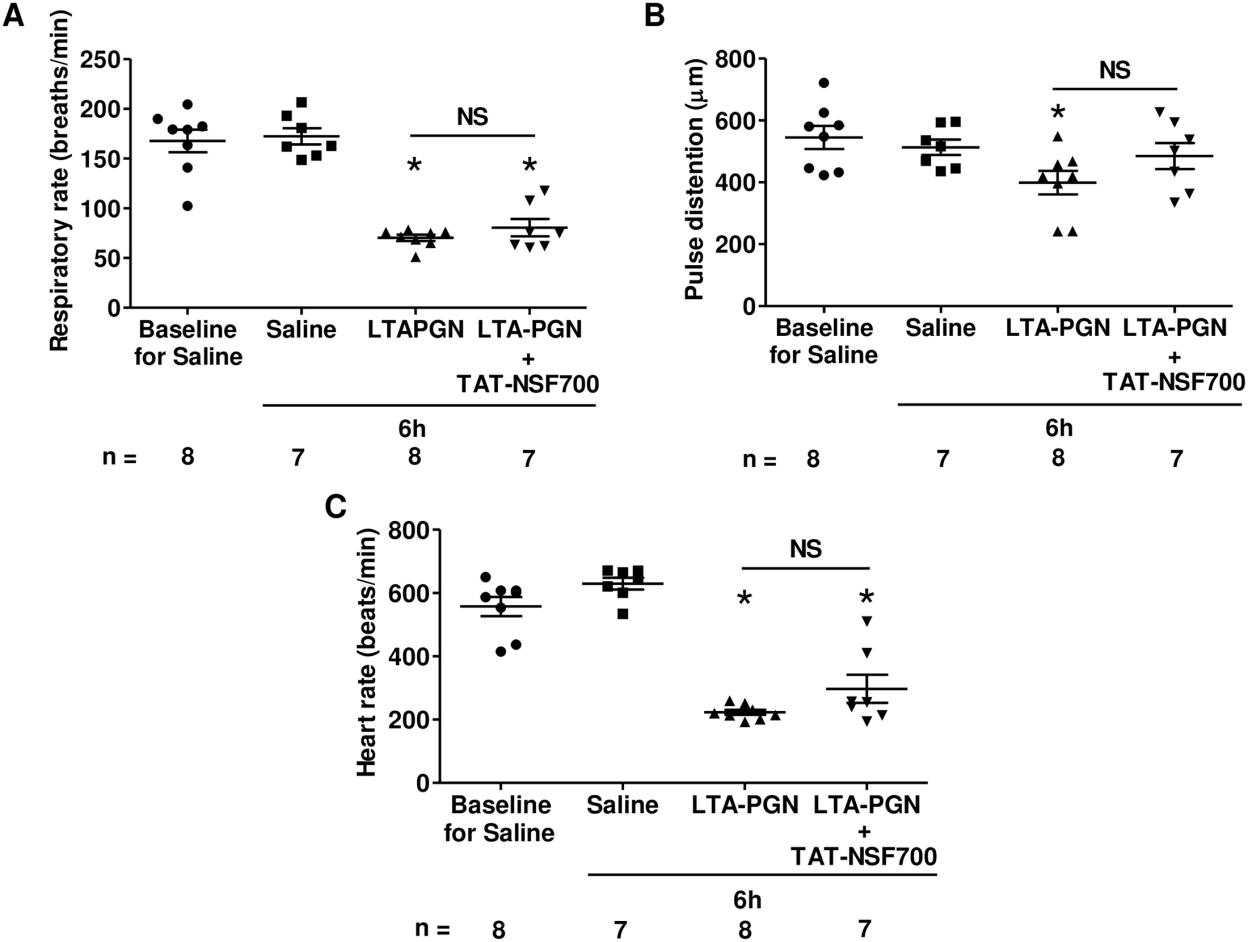
TAT-NSF700 effect on LTA-PGN induced respiratory rate, pulse distention and heart rate. [A] Respiratory rate, [B] pulse distention and [C] heart rate at baseline and 6 hours post LTA-PGN instillation are demonstrated (each column shows mean ± standard error; n = total number of mice; * *p* <0.05 *versus* Saline group).

In patients with severe sepsis and ARDS, alveolar filling with fluid, blood or inflammatory cells can cause significant shunt and gas exchange abnormality. However, in our animal model TAT-NSF700 prevented oxygen desaturation without significantly affecting the degree of LTA-PGN induced alveolar inflammation which was assessed by neutrophil recruitment, MPO activity, protein or other inflammatory cytokines such as KC or MIP-2 levels in BAL fluid (Table 1). Furthermore, in spite of decreases in respiratory rate in both LTA-PGN and TAT-NSF700 groups, oxygen saturation was still significantly better in the TAT-NSF700. The data suggests that hypoventilation is not a major factor responsible for LTA-PGN induced oxygen desaturation (Fig 2A).

**Table 1:** TAT-NSF700 effect on BAL fluid characteristics following pulmonary challenge.

	Control (n = 7)	LTA-PGN (n = 8)	LTA-PGN + TAT-NSF700 (n = 7)
**%PMN**	6.4 ± 5.8 (n = 6)	78.8 ± 18.1	74.0 ± 13.8 (n = 6)
**MPO (ng/ml)**	0.4 ± 0.2	1.8 ± 0.9	1.3 ± 0.4
**Total protein (μg/ml)**	166 ± 121.6	376 ± 119.8	327 ± 90.2
**KC (pg/ml)**	43 ± 18.2	2602 ± 85.4	2606 ± 81.9
**MIP-2 (pg/ml)**	22 ± 13.1	2145 ± 110.1	2144 ± 74.5

BAL fluid was obtained as described in Fig 1. Each value represents mean ± standard error; n = total number of mice. Compared to control group both LTA-PGN and TAT-NSF700 groups showed significant statistical difference (*p* < 0.05) in %PMN, total protein, KC and MIP-2 levels. Compared to LTA-PGN group, TAT-NSF700 had no statistical difference in all assessed parameters.

Pulse distention, a measurement of the diameter of pulsating neck vessel, reflects cardiac output in our experimental setting. Interestingly, there was a significant decrease in pulse distention in LTA-PGN group whereas no change in TAT-NSF700 group compared to control group (Fig 2B) in the setting of equally suppressed heart rate in both LTA-PGN and TAT-NSF700 groups (Fig 2C). This suggests that the LTA-PGN group had ventilation-perfusion mismatch due to decreased cardiac output and perfusion while this changes were not shown in TAT-NSF700 group potentially related to improving cardiovascular stability.

### *In Vitro* Studies

#### TAT-NSF700 attenuated LTA-PGN induced Ang-2 secretion in human pulmonary microvascular endothelial cells

**Fig 3:**
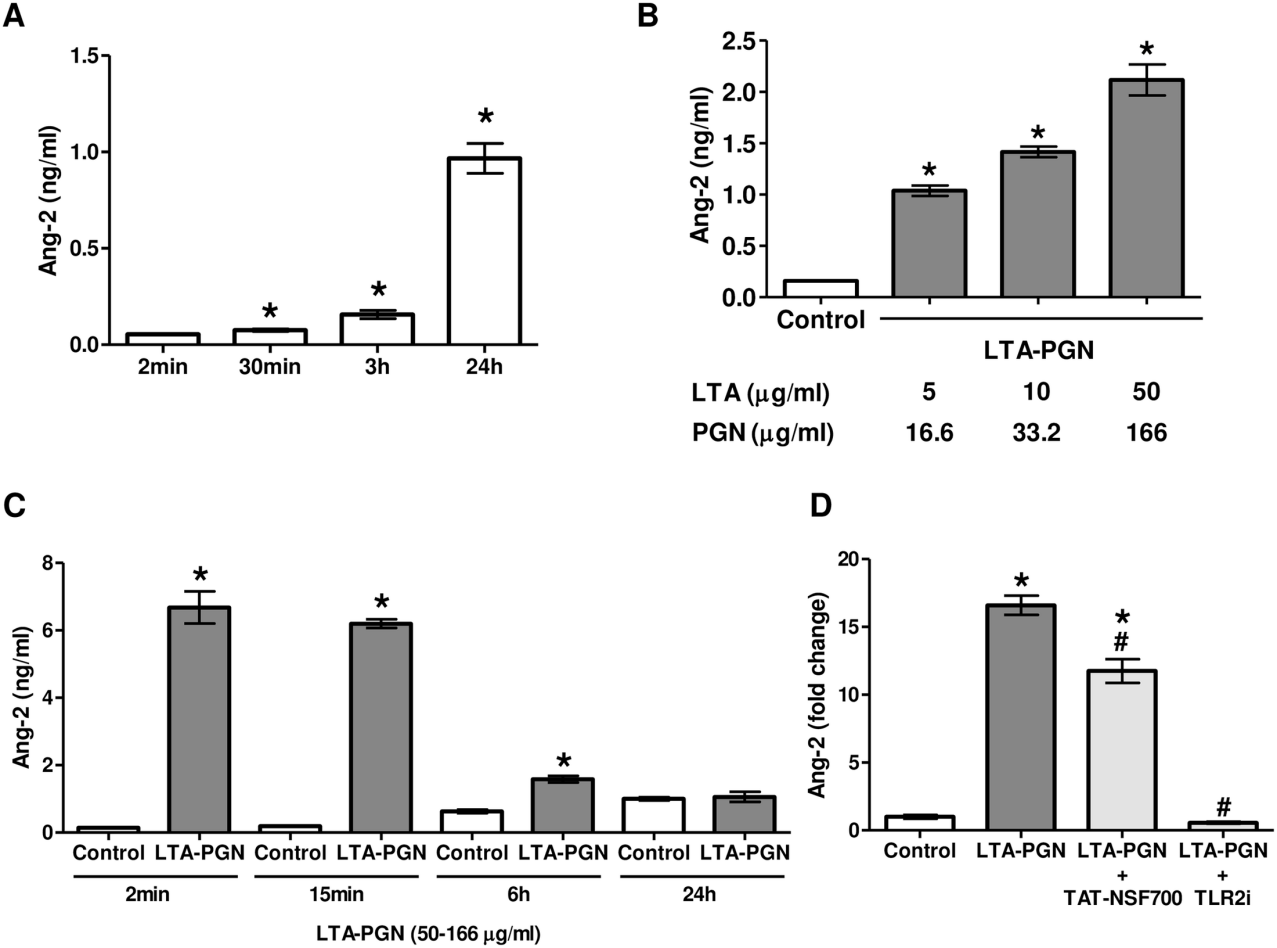
Characteristics of Ang-2 secretion in HPMEC-ST1.6R. Cells were grown to confluence in multi-well plates. They were serum starved overnight prior to the experiments. [A] Constitutive secretion of Ang-2 in HPMEC-ST1.6R culture medium was assessed at different time points by ELISA. [B] Ang-2 level changes in culture medium was assessed after 15min stimulation with LTA-PGN at different doses. [C] A time course of LTA-PGN induced and constitutive accumulation of Ang-2 was compared. [D] Cells were treated with serum free medium, TLR2 inhibitor (TLR2i, 15 μg/ml) or TAT-NSF700 (10 nM) for 1h followed by LTA-PGN (50–166 μg/ml) for 15 min. Ang-2 levels were measured in the culture medium (each column represents n = 3; mean ± standard error; * *p* <0.05 *versus* control group; # *p* <0.05 *versus* LTA-PGN group). All experiments were repeated at least 3 times independently.

To further investigate the dynamics of Ang-2 level change we cultured human pulmonary microvascular endothelial cells. At baseline, cells constitutively secreted Ang-2 shown by time dependent cumulative increase in cell culture medium (Fig 3A). LTA-PGN induced a concentration dependent increase in extra-cellular Ang-2 accumulation (Fig 3B). Compared to constitutive secretion, stimulated secretion was extremely rapid and potent. However, unlike in constitutive secretion where the accumulation increases with time, in stimulated secretion, the initial highly elevated accumulation decreased rapidly (Fig 3C). We further assessed downstream pathways of LTA-PGN induced Ang-2 secretion. Thus, cells were pre-treated with TAT-NSF700 or TLR2 receptor inhibitor for 1h prior to LTA-PGN stimulation. Culture medium was then collected 15min after LTA-PGN challenge. TLR2 receptor inhibitor completely and TAT-NSF700 partially inhibited LTA-PGN induced Ang-2 secretion (Fig 3D). The degree of the inhibitory effect of TAT-NSF700 was almost equal across the concentration range that we have tested, from 1nM to 10μM. This partial inhibitory effect of TAT-NSF700 is consistent with *in vivo* data. It suggests either the presence of an additional NSF independent LTA-PGN induced Ang-2 secretory pathway or a partial intracellular penetration of TAT-NSF700.

#### TAT-NSF700 attenuated LTA-PGN induced MLC phosphorylation in human pulmonary microvascular endothelial cells

**Fig 4:**
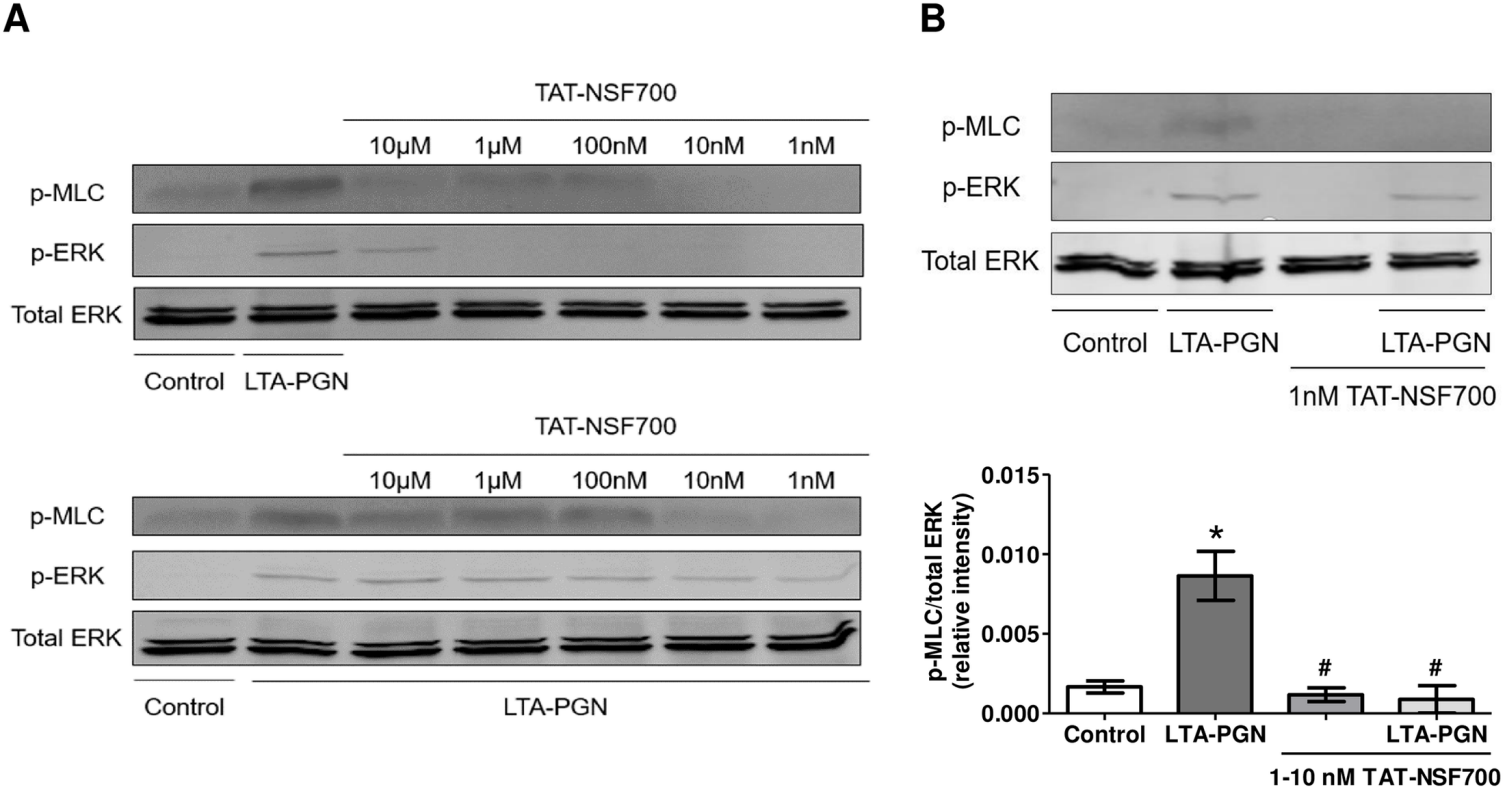
TAT-NSF700 effect on LTA-PGN induced MLC phosphorylation. [A] HPMEC-ST1.6R were cultured, pre-treated with serum free medium or TAT-NSF700 at different doses for 1h, then stimulated with LTA-PGN (50–166 μg/ml) for 15 min. [B] Cells were pre-treated with serum free medium or 1–10 nM TAT-NSF700 for 1h, then stimulated with LTA-PGN (50–166 μg/ml) for 15 min. Cell lysates were analyzed by western blot to assess ERK and MLC phosphorylation (each column represents n = 3; mean ± standard error; * *p* <0.05 *versus* control group; # *p* <0.05 *versus* LTA-PGN group).

Lastly, we investigated the mechanism of vascular stabilizing effect of TAT-NSF700 *in vitro*. Ang-2 is a well-known permeability inducing factor and its action is mediated by MLC phosphorylation [22]. We hypothesized that vascular stabilization afforded by TAT-NSF700 is by inhibition of Ang-2 secretion and/or independent inhibition of MLC phosphorylation by TAT-NSF700. At baseline, TAT-NSF700 significantly decreased MLC phosphorylation at low concentrations of 1-10nM whereas there was no change at higher concentrations of 100nM-10μM. This inhibitory effect of TAT-NSF700 at 1-10nM continued even after LTA-PGN stimulation (Fig 4). Notably, TAT-NSF700 at 10μM caused strong ERK phosphorylation in 15 min and significant gross cellular toxicity upon 24h incubation. Thus, we conclude that the inhibitory effect of TAT-NSF700 on LTA-PGN induced MLC phosphorylation is limited to low concentrations and that TAT-NSF700 itself may induces independent MLC phosphorylation at higher concentrations. However, further studies are needed to elucidate the mechanism by which TAT-NSF700, high concentrations, may itself induce MLC phosphorylation.

## Discussion

**Fig 5:**
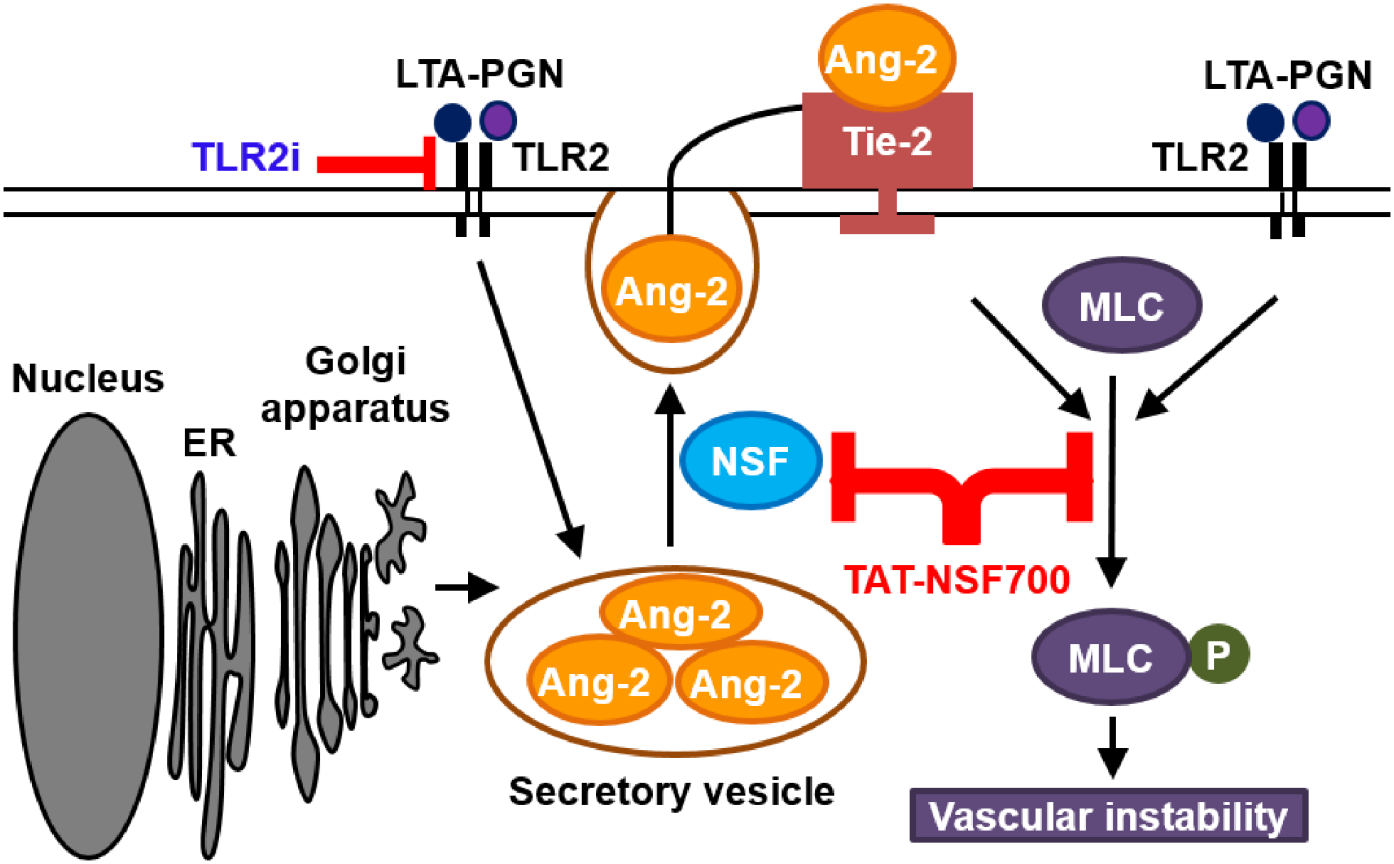
Schematic representation of the hypothesis of the mechanisms of oxygen saturation improvement by TAT-NSF700 following pulmonary challenge. TAT-NSF700 attenuates Ang-2 secretion and MLC phosphorylation potentially causing vascular stability leading to improved cardiovascular status, ventilation-perfusion mismatch and oxygen saturation.

Studies have demonstrated beneficial effects of TAT-NSF700 in different disease animal models including myocardial infarction, peritonitis and age-related arterial vasoconstriction [21, 23–25]. We assessed TAT-NSF700 effect on our mouse sepsis model, specifically on cardiovascular and respiratory changes, and on *in vitro* HPMEC-ST1.6R culture. We found that TAT-NSF700 attenuates Ang-2 secretion and MLC phosphorylation, although at a limited concentration range, inducing improved cardiovascular stability, decreased ventilation perfusion mismatch and improved oxygen saturation in LTA-PGN induced mouse sepsis (Fig 5).

In Fig 1A, we demonstrated lung homogenate Ang-2 concentration changes. LTA-PGN induced decreased lung tissue Ang-2 could be a result of increased secretion and/or decreased gene expression and protein synthesis. To answer this question, we measured Ang-2 plasma and lung tissue gene expression levels. Interestingly, both showed the same trend as that of lung tissue Ang-2 protein levels. Therefore, at first glance, we thought that LTA-PGN induced decreased lung tissue Ang-2 is secondary to decreased gene expression and secretion. However, *in vitro* data (Fig 3C) raised a possibility that there may have been a rapid secretion and depletion at an earlier time point. TAT-NSF700 induced significant inhibition of other inflammatory mediators such as KC and MIP-2 (data not shown) in plasma supported this idea that Ang-2 has a rapid peak and decrease compared to other late peaking mediators. Indeed, on our additional preliminary experiments of endpoints at earlier times such as <15min showed cases of extremely high plasma Ang-2 levels in LTA-PGN group. Taken together with the *in vitro* data, it is more reasonable to conclude that although Ang-2 gene expression and secretion is suppressed in LTA-PGN group by 6h, there already must have been excessive secretion, receptor binding and depletion of pre-synthesized storage pool at earlier time point.

The key finding of our study is that TAT-NSF700 dramatically improves oxygen saturation without any conventional therapies such as oxygen supplementation or mechanical ventilation. In the clinical setting, an oxygen saturation of 82% vs 95% (LTA-PGN vs TAT-NSF700 groups), in a patient would usually mean intubation vs no oxygen therapy. Given the risk, potential complications and healthcare burdens related to mechanical ventilation [26], this is an important finding and a new therapeutic strategy that could be further investigated for its clinical potential.

In spite of significant oxygen saturation improvement, TAT-NSF700 had no statistically significant effect on LTA-PGN induced alveolar inflammation. We think this is most likely secondary to improved vascular stability and ventilation perfusion matching given significant improvement in pulse distention in TAT-NSF700 group. However, we understand the limitation of non-invasive cardiopulmonary monitoring technique and do believe a gold standard invasive monitoring is necessary to validate our data.

We selected Ang-2 as a representative marker of sepsis/ARDS related pro-inflammatory mediator given its significant correlation with morbidity and mortality in clinical trials [13]. We demonstrated two distinctive patterns of Ang-2 secretion, constitutive and LTA-PGN stimulated. Although the WPB is an organelle known to contain various inflammatory mediators including Ang-2 [27] its presence is limited to endothelial cells from relatively large vessels, the diameter greater than 18 μm [28]. However, endothelial cells of capillary origin still have abundant vesicles mediating rapid and powerful secretion of inflammatory mediators. = Given the extremely rapid and potent Ang-2 secretion following LTA-PGN challenge, and a somewhat limited inhibitory effect of TAT-NSF700, it is possible that NSF independent, non-WPB secretory vesicles [29, 30] may be involved in the process. The possibility of incomplete intracellular penetration of TAT-NSF700 peptide should also be considered.

To better understand the mechanism of TAT-NSF700 induced cardiovascular stability we assessed MLC activation. MLC phosphorylation induces endothelial cell contraction, increased vascular permeability and decreased vascular tone [31–34]. It is known that Ang-2 induced permeability is mediated by MLC phosphorylation [22] and it is most likely mechanism of the beneficial effect of TAT-NSF700 on hemodynamic stability. However, future studies should address if there is a direct cause-effect relationship between Ang-2, MLC phosphorylation and permeability change in our experimental setting. Since NSF is a trafficking mediator molecule involved in the transport of various inflammatory mediators, MLC phosphorylation can be affected by molecules other than Ang-2. In addition, it is possible that NSF has a direct modulating effect on MLC phosphorylation.

## Conclusions

In conclusion, the data suggest a possible novel therapeutic approach in sepsis/ARDS by targeting NSF, a trafficking mediator molecule involved in the secretion of inflammatory mediators from endothelial cells and in the maintenance of vascular tone. TAT-NSF700 attenuated secretion of Ang-2 and MLC phosphorylation following pulmonary challenge, resulting in improved cardiovascular stability and ventilation perfusion matching and subsequently oxygen saturation, in the absence of any conventional sepsis treatment such as fluid resuscitation, vasopressor, oxygen supplementation or mechanical ventilation.
